# Chain-specific remodeling of the γδ T-cell TCR repertoire in preterm infants with cCMV: a gestational-age–stratified case–control study

**DOI:** 10.3389/fimmu.2026.1713669

**Published:** 2026-04-14

**Authors:** Peihui Liu, Rong Zou, Jie Zhao, Yu Zhang, Yun Liu, Powei Tang, Kai Wu, Xiujie Gao

**Affiliations:** 1 Department of Pediatrics, Shenzhen Maternity and Child Healthcare Hospital, Women and Children’s Medical Center, Southern Medical University, Shenzhen, Guangdong, China; 2 Department of Pharmacy, Shenzhen Maternity and Child Healthcare Hospital, Women and Children’s Medical Center, Southern Medical University, Shenzhen, Guangdong, China; 3 Neonatal Intensive Care Unit, Shenzhen Maternity and Child Healthcare Hospital, Women and Children’s Medical Center, Southern Medical University, Shenzhen, Guangdong, China; 4 School of Basic Medical Sciences, Guangdong Pharmaceutical University, Guangzhou, Guangdong, China

**Keywords:** CDR3, clonal diversity, congenital cytomegalovirus, preterm infants, TCR repertoire, TRD/TRG, V(D)J usage, γδ T cells

## Abstract

**Background:**

Congenital cytomegalovirus (cCMV) is a major cause of intellectual disability. γδ T-cells contribute to early-life antiviral defense, yet their T-cell receptor (TCR) repertoire in preterm cCMV remains incompletely defined.

**Methods:**

We profiled the γδ T-cell TCR repertoire in preterm infants allocated to three non-overlapping groups: Congenital CMV infection (cCMV) group (n=9), Acquired CMV infection (aCMV) group (n=10), and Preterm controls (CMV-negative) (n=7). RNA was extracted from RNAlater-stabilized heel-prick whole blood. Libraries were generated using constant-region–anchored primers for TRD/TRG, multiplex target enrichment, and index PCR, and sequenced on an Illumina platform (paired-end 150 bp). Repertoire features included CDR3 length distributions, V(D)J usage, VJ pairing, and clonal diversity. Nine lineage-associated rearrangements were quantified by intercalating dye–based qPCR.

**Results:**

TRD: the 25-aa CDR3 bin was less frequent in the cCMV group versus controls, and the cCMV group showed a lower Gini index, indicating less clonal dominance (greater evenness) than controls. TRG: cCMV and aCMV differed in a length-interval–dependent manner. V-gene usage shifted by group (TRAV29/DV5 and TRAV38-2/DV8 in TRD; TRGV2/4/8/9 in TRG), and VJ pairings were more frequent in cCMV than in aCMV. The cCMV group harbored more “specific” clonotypes than controls (P = 0.020). qPCR showed higher expression of Dδ2–Dδ3, Vδ2–Jδ1, Vγ–Jγ1.3/2.3 in cCMV; the mixed Vγ9+Vγ11–Jγ1.1/2.1 signal was also elevated but requires deconvolution.

**Conclusions:**

cCMV is associated with chain-specific remodeling of the γδ T-cell repertoire in preterm infants. These data may inform molecular risk stratification in neonatal CMV. Findings should be interpreted in light of small sample size and RNA-level profiling.

## 1 Introduction

Congenital cytomegalovirus (cCMV) infection is one of the most common intrauterine infections worldwide, and it is also an important cause of neonatal nervous system sequelae (such as hearing impairment, cerebral palsy, intellectual disability, etc.) (1, 2). The risk of congenital infection caused by primary infection of maternal CMV is 30% ~ 40%, while the risk of non-primary infection is only 1.1% ~ 1.7% (3). Premature infants are more susceptible to CMV infection because of immature immune system, and the prognosis after infection is often worse (4). The immune response to CMV involves a variety of immune cells, including natural killer (NK) cells, macrophages, αβ T cells and γδ T cells. γδ T cells are a kind of unconventional T cells. Compared with traditional αβ T cells, γδ T cells have unique antigen recognition mechanism and immune function (5–7). γδ T cells can directly recognize non-peptide antigens without the participation of antigen presenting cells, so they can respond to infection quickly (8). In addition, γδ T cells can kill infected cells by releasing cytokines (such as IFN-γ and TNF-α) and cytotoxic molecules (9). A hallmark of the γδ T cell response to human CMV is the selective expansion of Vδ2-negative (predominantly Vδ1+) γδ T cells, leading to a shift in the Vδ2+/Vδ2− ratio. This phenomenon has been observed consistently across diverse clinical contexts, including solid organ and hematopoietic stem cell transplant recipients, pregnant women, neonates, and healthy seropositive individuals (10).

The TCR of γδ T cells is composed of TCRγ and TCRδ, and its gene rearrangement process is similar to that of αβ T cells, involving the combination of V, D and J gene fragments (10, 11). During individual development, γδ T cells emerge from the thymus and are directly deployed to peripheral tissues for *in situ* immune responses. Childhood antigen exposure drives the functional evolution and tissue compartmentalization of γδ T cells, leading to age-dependent immune responses (12). It was found that in sepsis, CD83 was expressed on Vγ9Vδ2 T cells activated by premature infants, but not in adults. At the same time, the mature map of γδ T cells after premature delivery shows their phenotypic diversity in infection. This suggests that γδ T cells may play an important role in the early immune defense (13). Chen et al. followed up a newborn with congenital cytomegalovirus infection, and found that the expression levels of perforin and granzyme B secreted by γδ T cells increased with the extension of hospital stay, which indicated that the immune environment faced by intrauterine infection and postnatal infection was different (14).

The expression profile of TCR gene can reflect the clonal composition and functional status of γδ T cells, so it is very important to study the expression characteristics of TCR gene for understanding the role of γδ T cells in immune response. In this context, a clonotype is defined as a unique CDR3 nucleotide (or amino acid) sequence arising from a specific V-(D)-J rearrangement, identified via high-throughput sequencing and annotated according to IMGT standards. V-gene segment nomenclature follows the IMGT/Heilig–Tonegawa system (e.g., TRGV8, TRDV1, TRDV2). At present, the research on CMV infection of neonatal γδ T cells mainly focuses on the subtype distribution of αβ T cells and TCR expression. However, there are relatively few studies on the expression characteristics of TCR gene in γδ T cells infected by CMV in premature infants. Due to the immature immune system, the composition and function of γδ T cells in premature infants may be different from those in full-term infants. Therefore, in-depth study on the expression characteristics of TCR gene in γδ T cells infected by premature infants with cCMV will help to better understand the immunopathological mechanism of cCMV infection and provide a basis for developing immunotherapy strategies for premature infants with cCMV infection. The purpose of this study is to analyze the expression characteristics of TCR gene in γδ T cells of premature infants infected with cCMV by highly sensitive pre-amplification real-time fluorescence PCR and TCR immunohistochemical library technology, to explore its role in cCMV infection, and to try to establish a graded diagnosis and evaluation model of cCMV infection based on molecular markers. Specifically, we hypothesized that: (i) cCMV would induce chain-specific remodeling of the γδ TCR repertoire, with distinct alterations in TRD and TRG chains compared to postnatally acquired CMV and CMV-negative controls; (ii) V-gene usage patterns, particularly TRGV8 enrichment, would mirror the fetal γδ T cell response described for CMV; and (iii) diversity indices and clonotype counts would differ by infection timing (*in utero* vs. postnatal), reflecting distinct immune pressures.

## 2 Materials and methods

### 2.1 Research objects

In this study, preterm infants with a gestational age of 24 + 0 to 36 + 6 weeks were enrolled from the Department of Neonatology at Shenzhen Maternity and Child Healthcare Hospital, a tertiary care center, between October 2022 and December 2024. Whole blood samples were collected via heel prick. In accordance with the international consensus recommendations (15), participants were stratified into three non-overlapping groups based on CMV infection status: (1) the congenital CMV (cCMV) group (CMV DNA positive ≤21 days of life); (2) the acquired CMV (aCMV) group (CMV DNA positive >21 days of life); and (3) the preterm controls group (CMV DNA negative throughout hospitalization). The study protocol was approved by the Institutional Review Board of Shenzhen Maternity and Child Healthcare Hospital (Approval No. SFYLS [2022]070) and conducted in accordance with the Declaration of Helsinki. Written informed consent was obtained from the parents or legal guardians of all participating infants.

### 2.2 Definition criteria for each group

#### 2.2.1 Congenital CMV group

CMV infection status: CMV DNA positive in urine within 3 weeks after birth, indicating congenital infection.

#### 2.2.2 Acquired CMV group

CMV infection status: CMV DNA positive in urine after 3 weeks of age, indicating acquired (postnatal) infection, documented exposure to postnatal transmission sources (such as CMV-positive breastfeeding, blood transfusion, etc.).

#### 2.2.3 Preterm controls group (CMV-negative)

CMV infection status: CMV DNA negative in urine at all time points during hospitalization.

Exclusion criteria: Major chromosomal abnormalities; Other confirmed congenital infections (TORCH panel excluding CMV); Other serious infections (such as bacterial meningitis, sepsis, etc.); Congenital immunodeficiency syndromes unrelated to CMV; Maternal antiviral therapy during pregnancy (for cCMV cases); Inadequate sample volume (<50 μL) for RNA analysis.

### 2.3 Sample collection

After skin antisepsis and warming of the heel, peripheral whole blood (200 µL) was collected via heel prick into EDTA microtainers within 24 hours after CMV diagnosis confirmation for infants in the cCMV group and aCMVgroup, while for those in the preterm controls group, collection was performed within the neonatal period. The blood was then mixed by gentle inversion in a tube containing RNAlater, stored at 4 °C for ≤24 hours, and subsequently transferred to −80°C pending RNA extraction.

### 2.4 TCR group library analysis

Total RNA was extracted from RNAlater-stabilized neonatal whole blood (200 µL) using the HiPure Blood RNA Mini Kit (Magen, Cat. No. R4161-02) with scaled RBC-lysis and leukocyte-enrichment steps, following the manufacturer’s instructions. RNA quantity was measured with a Qubit 4.0 Fluorometer (Thermo Fisher Scientific). For TCR repertoire libraries, 100 ng total RNA (or the maximum available amount when <100 ng) was used as input for cDNA synthesis with constant-region–anchored primers (TRD/TRG), multiplex target enrichment PCR, and index PCR, prior to QC by gel electrophoresis and fluorometric quantification. Pooled libraries were sequenced on an Illumina platform (PE150). Specifically, cDNA was synthesized using reverse primers anchored in the TRDC and TRGC constant regions; the full primer panel is provided in **Table 1**. Libraries were validated by Agilent 2100 Bioanalyzer (expected fragment size: 300–500 bp) and Qubit fluorometric quantification (acceptance threshold: >2 nM). Each sample was sequenced to a minimum depth of 1 × 10^6^ raw paired-end reads.

**Table 1:** Primer sets for PCR detection of TCRD (TRD) and TCRG (TRG) rearrangements.

Target rearrangement	Primer ID	Sequence (5′→3′)
TCRD (TRD)		
Dδ2–Dδ3	dδ2-for	CGGGTGGTGATGGCAAAGTGCC
	dδ3-rev	GAAATGGCACTTTTGCCCCTGCAG
Dδ2–Jδ1	dδ2-for	CGGGTGGTGATGGCAAAGTGCC
	jδ1-rev	GAGTTACTTACTTGGTTCCAC
Vδ1–Jδ1	vδ1-for	ACTCAAGCCCAGTCATCAGTATCC
	jδ1-rev	GAGTTACTTACTTGGTTCCAC
Vδ2–Jδ1	vδ2-for	ACCTGGCTGTACTTAAGATACTTGC
	jδ1-rev	GAGTTACTTACTTGGTTCCAC
Vδ5–Dδ3	vδ5-for	ACCCTGCTGAAGGTCCTACAT
	dδ3-rev	TGGGACCCAGGGTGAGGATAT
TCRG (TRG)		
Vγ1f + Vγ10–Jγ1.1/2.1	vγ1f-for	GGAAGGCCCCACAGRTCTT
	vγ10-for	AGCATGGGTAAGACAAGCAA
	jγ1.1/2.1 (JP1/2)-rev	AGGCGAAGTTACTATGAGCY
	trgj1-rev	TGTGACAACMAGTGTTGTTC
Vγ9 + Vγ11–Jγ1.1/2.1	vγ9-for	CGGCACTGTCAGAAAGGAATC
	vγ11-for	CTTCCACTTCCACTTTGAAA
	jγ1.1/2.1 (JP1/2)-rev	AGGCGAAGTTACTATGAGCY
Vγ (multiple)–Jγ1.3/2.3	trgv1-for	TCTTCCAACTTGGAAGGGRG
	jγ1.3/2.3-rev	GAGAAACCGTCACCTTGTTGTG
Vγ (multiple)–Jγ1.2	trgv1-for	TCTTCCAACTTGGAAGGGRG
	jγ1.1/2.1 (JP1/2)-rev	AGGCGAAGTTACTATGAGCY
Housekeeping control		
GAPDH	gapdh-for	TGCAACCGGGAAGGAAATGA
	gapdh-rev	GCATCACCCGGAGGAGAAAT

Degenerate bases follow IUPAC codes (R = A/G, Y = C/T, M = A/C, N = any). Group headers are for readability. All sequences are shown 5′→3′.

### 2.5 Real-time fluorescence PCR with high sensitivity preamplification

Using the same RNA extraction batch as in Section 2.4, the residual total RNA (not used for repertoire library construction) was carried forward to the preamplification-based real-time PCR workflow to minimize variability introduced by repeat sampling and extraction. Briefly, total RNA had been isolated from RNAlater-stabilized neonatal whole blood (200 µL) using the HiPure Blood RNA Mini Kit (Magen, Cat. No. R4161-02) performed according to the manufacturer’s instructions. RNA concentration was measured using a Qubit 4.0 Fluorometer (Thermo Fisher Scientific). For relative quantification assays, 5 µL of the residual RNA from each sample was used as input for downstream cDNA synthesis, followed by preamplification and SYBR Green–based real-time fluorescence PCR.

For the first-round PCR (preamplification), reactions were prepared in a total volume of 25 µL, containing cDNA template, PCR Mix, and gene-specific primers. Cycling conditions were 95 °C for 5 min, followed by 15 cycles of 95 °C for 30 s, 55 °C for 30 s, and 72 °C for 30 s, with a final extension at 72 °C for 10 min. For the second-round amplification (SYBR Green qPCR), reactions were also prepared in a total volume of 25 µL, containing diluted first-round PCR products as template, SYBR Green intercalating dye–based qPCR Mix, and the corresponding primers. The cycling program was 95 °C for 5 min, followed by 35 cycles of 95 °C for 30 s, 55 °C for 30 s, and 72 °C for 30 s. Fluorescence was collected during amplification.

Nine γδ T-cell differentiation–associated rearrangement targets were quantified, including Dδ2–Dδ3, Dδ2–Jδ1, Vδ1–Jδ1, Vδ2–Jδ1, Vδ2–Dδ3, Vγ1f+Vγ10–Jγ1.1/2.1, Vγ9+Vγ11–Jγ1.1/2.1, Vγ’s–Jγ1.3/2.3, and Vγ’s–Jγ1.2. Relative expression levels were calculated using the 2^-ΔΔCt method with GAPDH as the internal reference gene (**Table 1**).

### 2.6 Data analysis

The sequencing data were analyzed by MiXCR software [16](version 4.x; https://github.com/milaboratory/mixcr), including sequence alignment, V(D)J gene fragment identification, clonal identification and so on. The MiXCR pipeline consisted of: (1) align (species: hsa; starting material: rna; receptor chains: TRD, TRG; minimum quality score: 20); (2) assemblePartial (two rounds); (3) assemble (minimum clone count: 2 reads); and (4) exportClones. Non-productive rearrangements (containing stop codons or out-of-frame junctions) were filtered out prior to downstream analysis. A clonotype was defined as a unique CDR3 amino acid sequence arising from a specific V-(D)-J rearrangement, annotated according to IMGT nomenclature. The clone diversity index (such as Shannon entropy index and Simpson index) and the frequency of V(D)J gene fragment were calculated for each sample. Statistical analyses were performed using R (version 4.X). For all repertoire metrics (CDR3 length distributions, V-gene usage frequencies, VJ pairing frequencies, diversity indices, and clonotype counts), group comparisons were performed using Kruskal–Wallis tests across all three groups; where significant (P < 0.05), Dunn’s *post-hoc* pairwise comparisons were applied with Benjamini–Hochberg false discovery rate (FDR) correction. Adjusted P-values are reported as q-values throughout. Where appropriate, effect sizes (Cliff’s delta for pairwise comparisons; eta-squared for Kruskal–Wallis) are also reported. Descriptive statistics are presented as median (interquartile range, IQR) unless otherwise stated. Significance thresholds: ns, P ≥ 0.05; *, P < 0.05; **, P < 0.01; ***, P < 0.001.

## 3 Results

### 3.1 Basic information of clinical data

The clinical situation of each group is shown in **Table 2**. The cCMV group comprised 9 preterm infants (median gestational age: 31 + 3 weeks, range: 25 + 2–35 + 2 weeks; median birth weight: 1,310 g, range: 790–2,490 g). The first positive CMV DNA PCR was obtained at a median DOL of 10.0 (range: 2–17), with median CMV viral load of 3.89 × 10^4^ copies/mL (range: 1.88 × 10³ – 1.58 × 10^6^). The aCMV group comprised 10 preterm infants (median gestational age: 31 + 4 weeks, range: 24 + 2–36 + 3 weeks; median birth weight: 905g, range: 580–3,000 g), the first positive CMV DNA PCR was obtained at median DOL 38.5 (range: 28–62), with median CMV viral load of 6.41 × 10^3^ copies/mL (range: 2.02 × 10³ – 7.31 × 10^5^). The Preterm controls group comprised 7 infants (median gestational age: 31 + 5 weeks, range: 25 + 3–34 + 2 weeks; median birth weight: 1,110 g, range: 630–1,980 g), with blood collection at median DOL 17.0 (range: 7–27).

**Table 2:** Clinical characteristics of the participants.

Group	No.	Sex	Gestational age (weeks)	Birth weight (g)	Day of Life (DOL) of the positive PCR	Viral load in urine (copies/mL)	Clinical manifestations consistent with CMV infection
cCMV	1	M	30^+4^	1017	12	3.89 × 10^4^	intrauterine growth restriction
	2	M	35^+2^	1310	2	6.25 × 10^5^	ventriculomegaly, intracerebral calcifications, sensorineural hearing loss,hepatitis,petechiae
	3	M	35^+2^	2350	2	1.58 × 10^6^	hepatitis,cholestasis, mild sensorineural hearing loss,thrombocytopenia
	4	F	30^+5^	1540	13	1.88 × 10³	—
	5	M	33^+6^	2490	17	1.02 × 10^4^	—
	6	F	31^+5^	1750	10	5.73 × 10^4^	cholestasis
	7	F	27^+0^	920	11	1.76 × 10^4^	mild hepatomegaly,mild hepatitis
	8	M	25^+2^	790	10	2.03 × 10^5^	—
	9	F	31^+3^	1160	9	1.42 × 10^4^	—
aCMV	1	M	36^+0^	3000	30	2.02 × 10^3^	—
	2	F	24^+2^	580	41	7.31 × 10^5^	mild hepatomegaly,mild hepatitis
	3	F	36^+3^	2700	28	6.78 × 10³	—
	4	M	31^+5^	815	33	3.13 × 10³	—
	5	M	27^+3^	699	62	1.99 × 10^4^	—
	6	M	28^+3^	690	55	3.69 × 10^5^	hepatitis,cholestasis
	7	F	32^+0^	1090	45	4.34 × 10³	—
	8	F	31^+3^	930	40	3.56 × 10³	mild hepatitis
	9	F	33^+1^	1210	33	6.03 × 10³	—
	10	M	29^+4^	880	37	4.44 × 10^5^	—
Control	1	M	31^+5^	1980	—	Negative	—
	2	F	32^+2^	1110	—	Negative	—
	3	F	25^+3^	690	—	Negative	—
	4	M	26^+0^	860	—	Negative	—
	5	M	29^+2^	630	—	Negative	—
	6	F	34^+2^	1740	—	Negative	—
	7	M	31^+5^	1660	—	Negative	—

cCMV, congenital cytomegalovirus infection; aCMV, acquired (postnatal) cytomegalovirus infection; M,male; F, female; — , none. Gestational age expressed as weeks^+^days. Viral load presented as cytomegalovirus DNA copies per milliliter.

### 3.2 Length distribution characteristics of TRG/TRD CDR3

**Figure 1:**
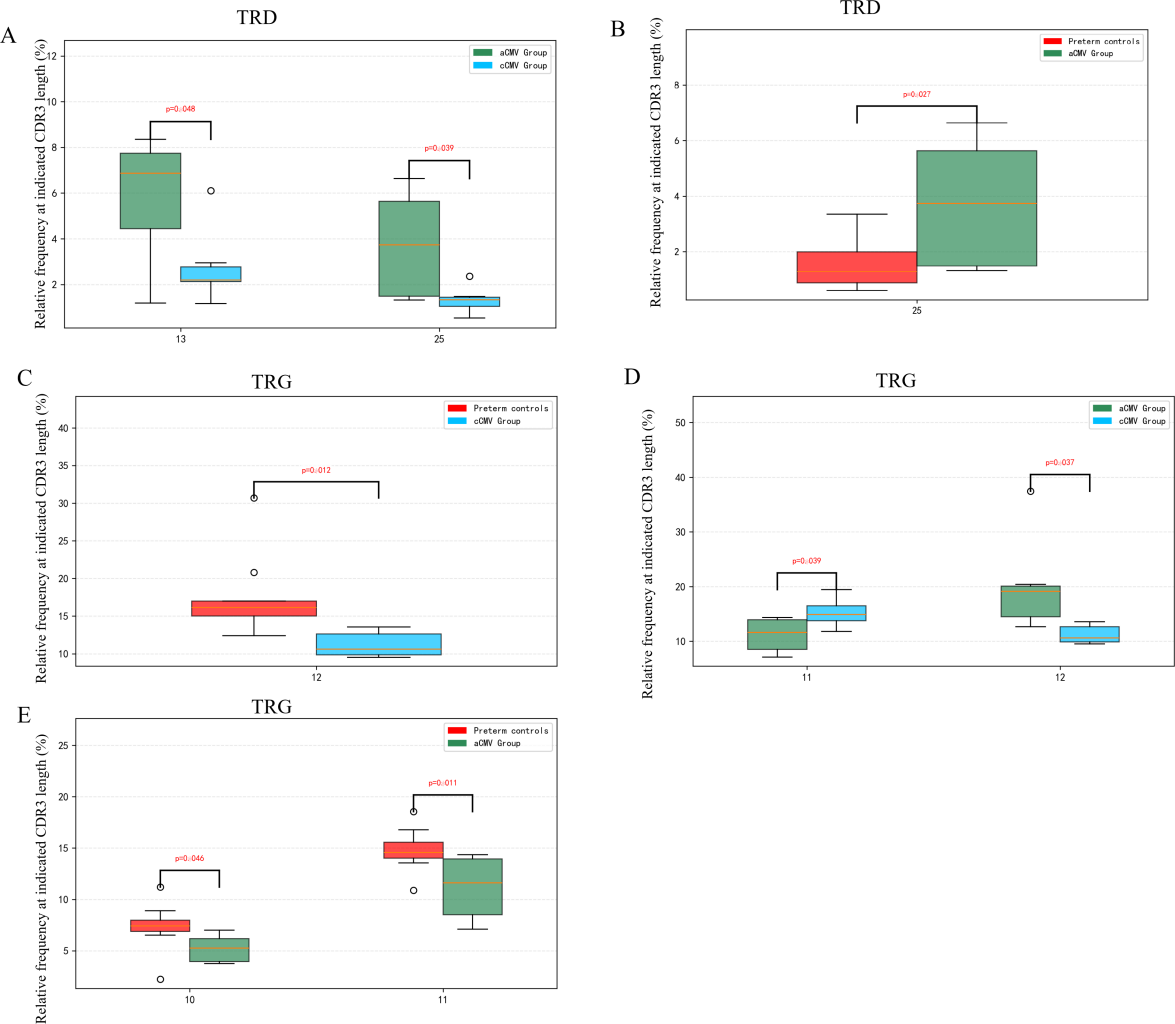
CDR3-length–specific differences in TRD (TCRδ) and TRG (TCRγ) repertoires across groups. **(A, B)** TRD chain (selected CDR3 lengths: 10 aa and 25 aa); **(C–E)** TRG chain (selected CDR3 lengths: 10–12 aa). For each length bin, box-and-whisker plots show the per-infant relative frequency of productive clonotypes at that exact CDR3 length (normalized within chain). All three groups are displayed in each panel: Early-detection (cCMV) group (n = 9, red), Preterm controls (CMV-negative) (n = 7, green), and Late-detection (aCMV) group (n = 10, blue). Dots represent individual infants; boxes indicate median and IQR; whiskers denote 1.5×IQR. All comparisons were performed using Kruskal–Wallis tests across the three groups; significant pairwise comparisons (Dunn’s *post-hoc*, Benjamini–Hochberg FDR-adjusted) are indicated by brackets. ns, P>0.05; *P<0.05; **P<0.01; ***P<0.001.

In the analysis of TRD chain, there length-interval–dependent differences were observed. The aCMV group showed a higher frequency at the 13-aa bin (median = 6.87%) and 25-aa bin (median = 3.73%) compared to the cCMV group (**Figure 1A**; P < 0.05). The aCMV group also differed from preterm controls at 25 aa (**Figure 1B**; P < 0.05). In the analysis of TRG chain, length-interval–dependent differences were observed. The cCMV group showed a higher frequency at the 11-aa bin (median = 14.87%) but lower frequency at the 12-aa bin (median = 10.62%) compared to the aCMV group (**Figure 1D**; P < 0.05). The cCMV group also differed from preterm controls at 12 aa (**Figure 1C**; P < 0.05), and the aCMV group differed from controls at 10 aa and 11 aa (**Figure 1E**; P < 0.05).

### 3.3 V gene

**Figure 2:**
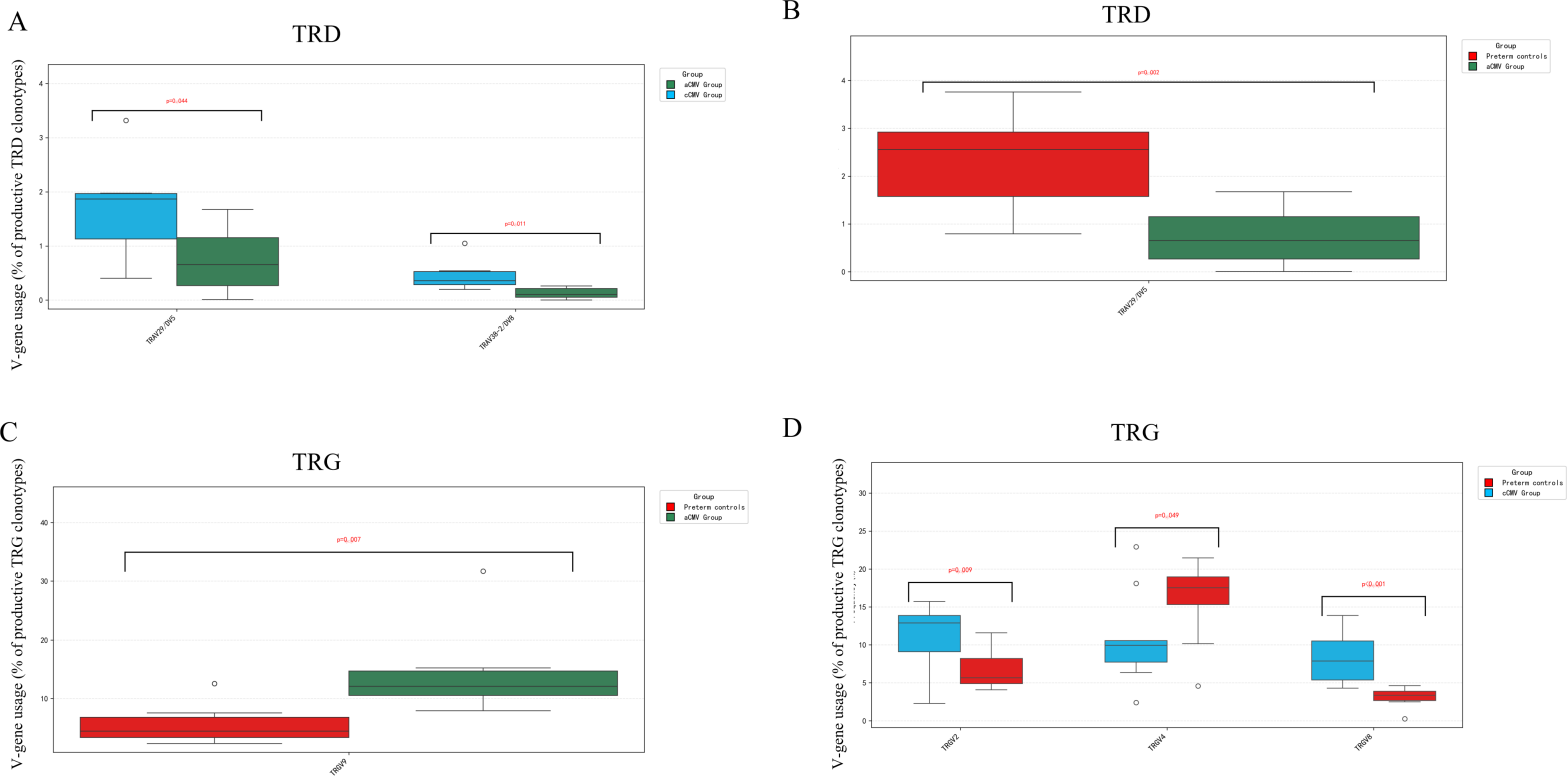
V-gene usage in TRD and TRG γδ-TCR repertoires across groups. **(A, B)** TRD V-genes; **(C, D)** TRG V-genes. All three groups are displayed in each panel with consistent color coding as in **Figure 1**. Box-and-whisker plots show the per-infant usage frequency expressed as a percentage of productive clonotypes within the corresponding chain. Kruskal–Wallis tests with Dunn’s *post-hoc* pairwise comparisons (FDR-adjusted); significance brackets shown for q < 0.05.

TRD V-gene usage: Within the cCMV group, the usage frequency of TRAV29/DV5 was significantly higher (median = 1.87%) and TRAV38-2/DV8 lower (median = 0.36%) compared to controls (**Figure 2A**; q < 0.05). Preterm controls showed higher TRAV29/DV5 frequency than the aCMV group (**Figure 2B**; P < 0.05).

TRG V-gene usage: TRGV9 usage was lower in preterm controls (median = 4.44%) than in the aCMV group (median = 12.08%; **Figure 2C**; P < 0.05). Notably, TRGV8 usage was significantly higher in the cCMV group (median = 7.86%) compared to preterm controls (median = 3.35%; **Figure 2D**; P < 0.05), while TRGV2 was lower and TRGV4 was higher in the cCMV group relative to controls.

### 3.4 VJ gene

**Figure 3:**
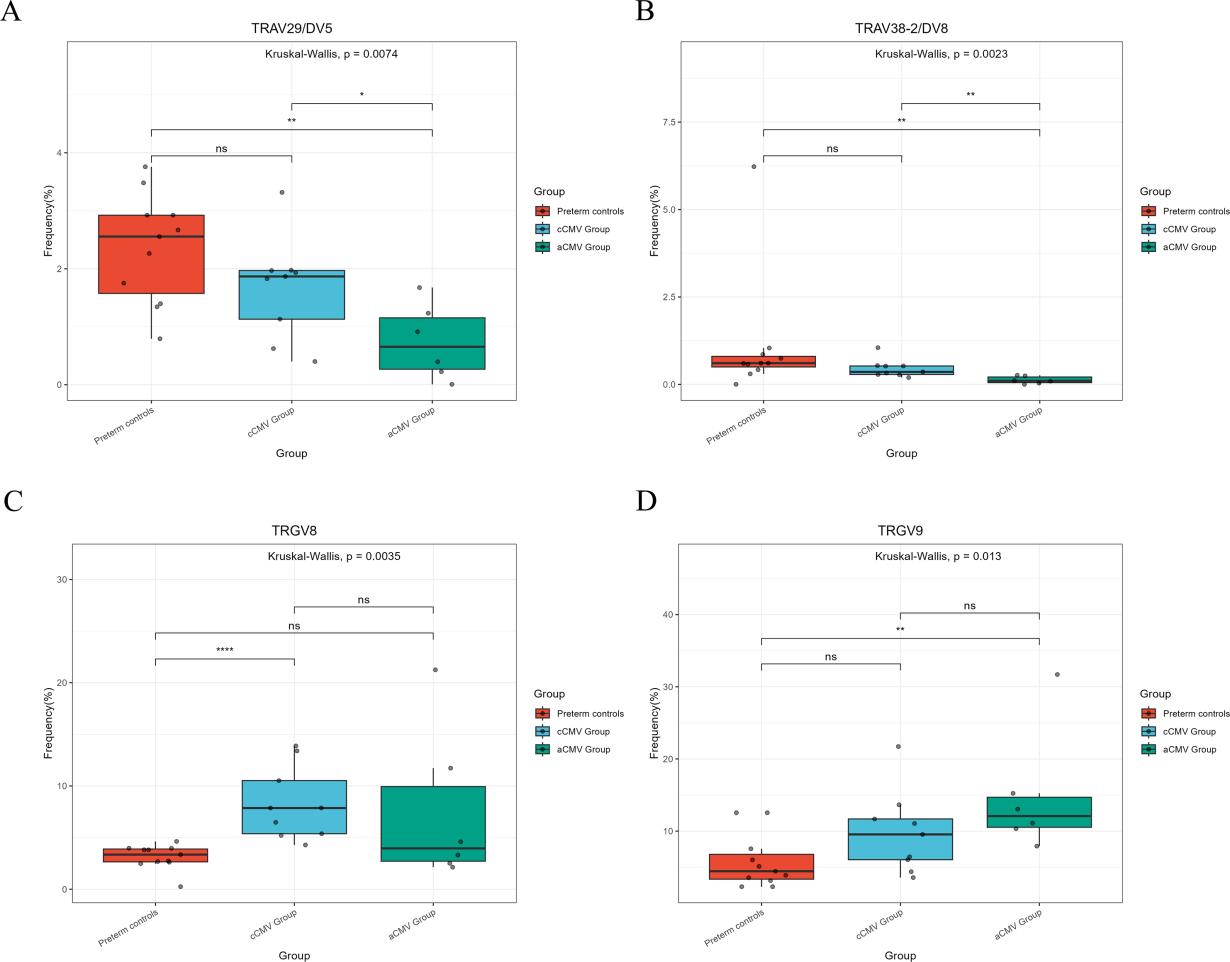
V–J pairing frequencies in TRD and TRG γδ-TCR repertoires across groups. **(A, B)** TRD V–J pairings (as labeled on the x-axis). **(C, D)** TRG V–J pairings (as labeled). For each pairing, box-and-whisker plots show the per-infant usage frequency expressed as the percentage of productive clonotypes within the corresponding chain. Groups: Congenital CMV infection (cCMV) group (n=9), Preterm controls (CMV-negative) (n=7), and Acquired CMV infection (aCMV) group (n=10). Dots indicate individual infants; boxes show median and IQR; whiskers denote 1.5×IQR.Between-group comparisons used Kruskal–Wallis tests with Dunn’s *post-hoc* pairwise comparisons. *P<0.05; **P<0.01; ***P<0.001.

Analysis of V–J pairing frequencies revealed group-specific preferences. TRD results showed that the frequency of TRAV29/DV5-TRDJ1 pairing in preterm controls and the cCMV group was significantly higher than in the aCMV group (**Figure 3A**, P < 0.05). Similarly, the frequency of TRAV38-2/DV8-TRDJ1 in preterm controls (CMV-negative) and cCMV group was also significantly higher than the aCMV group (**Figure 3B**, P < 0.05). The results of TRG showed that the frequency of using TRGV8 in cCMV group was significantly higher than in preterm controls (CMV-negative) (**Figure 3C**, P < 0.05). The frequency of TRGV9 in aCMV group was also significantly higher than in preterm controls (CMV-negative) (**Figure 3D**, P < 0.05). cCMV group, aCMV group and preterm controls (CMV-negative) group showed obvious preference in the use of specific VJ gene combinations of TRD and TRG chains, indicating that the TCR diversity of γδ T cells in different groups was affected by different factors. The high frequency of specific TRD VJ combinations such as TRAV38-2/DV8/TRDJ1 in cCMV group may reflect the immune response to the specific antigen of cCMV infection. However, the difference in specific TRG VJ combinations between the preterm controls (CMV-negative) may be related to the unique immune development status of premature infants.

### 3.5 Diversity TRD

**Figure 4:**
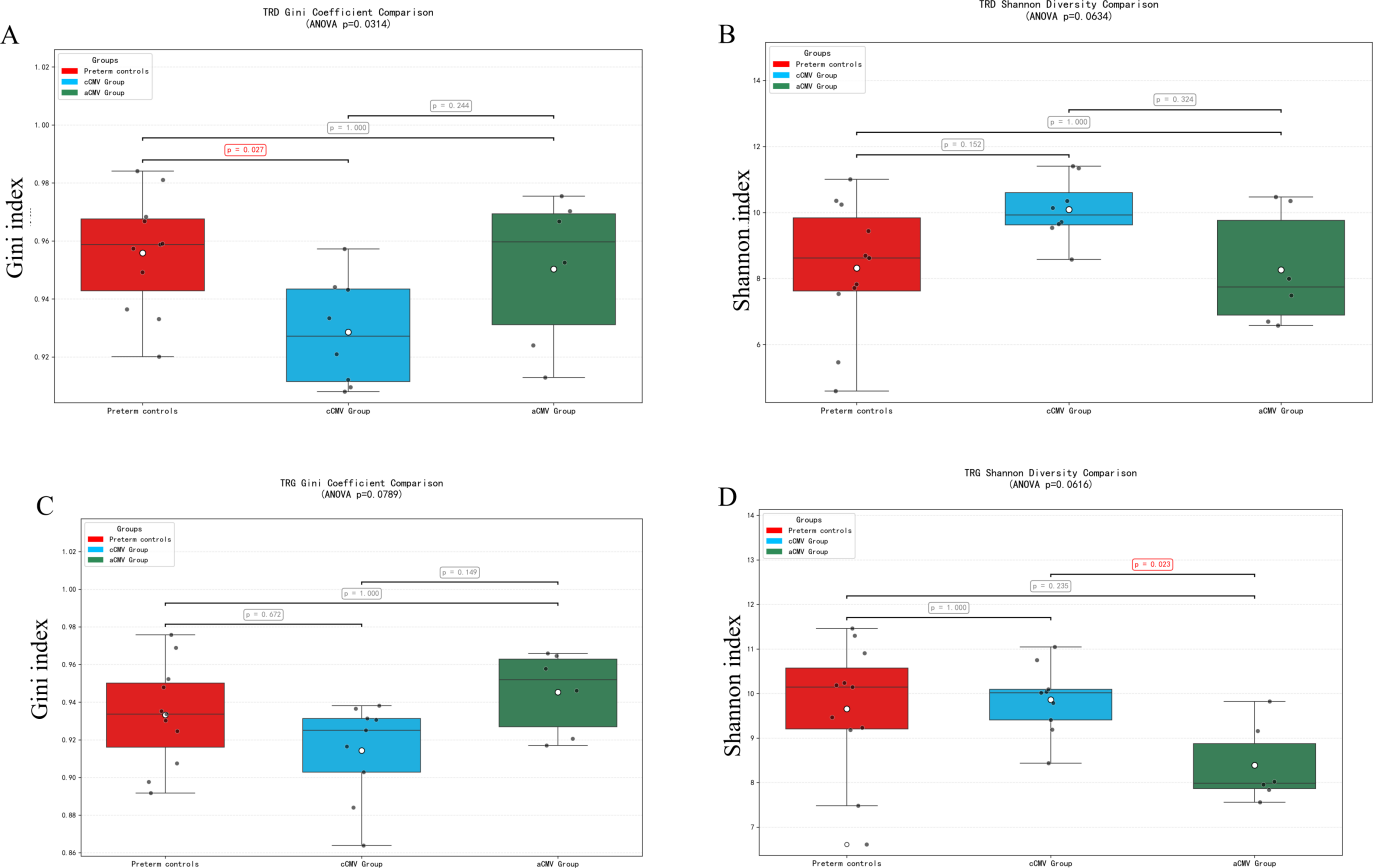
Gini and Shannon diversity of TRD and TRG γδ TCR repertoires across groups. **(A)** TRD—Gini index; **(B)** TRD—Shannon index; **(C)** TRG—Gini index; **(D)** TRG—Shannon index. Box–whisker plots show per-infant diversity metrics for the Congenital CMV infection (cCMV) group (n=9), Preterm controls (CMV-negative) (n=7), and the Acquired CMV infection (aCMV) group (n=10). Dots represent individual infants; boxes indicate median and IQR; whiskers denote 1.5×IQR. Group comparisons used Kruskal–Wallis tests with Dunn’s *post-hoc* pairwise comparisons; P values were FDR-adjusted (reported as q). Interpretation: higher Gini = greater clonal concentration (lower evenness); higher Shannon = greater repertoire diversity. ns, P>0.05; *P<0.05; **P<0.01; ***P<0.001.

The differences of Gini index and Shannon index between preterm controls (CMV-negative) and cCMV and aCMV infection were compared. The Gini index was significantly higher in preterm controls (median = 0.9337, IQR: 0.9161–0.9501) than in the cCMV group (median = 0.9250, IQR: 0.9029–0.9313; Dunn’s P = 0.027), indicating greater clonal concentration (a few dominant clones) in controls versus more evenly distributed clones in cCMV (**Figure 4A**). The Shannon index did not differ significantly among the three groups for TRD (Kruskal–Wallis P > 0.05; **Figure 4B**).

The Gini index did not differ significantly among groups for TRG (**Figure 4C**; P > 0.05). However, the Shannon index was significantly lower in the aCMV group (median = 7.9874) than in the cCMV group (median = 10.0171; Dunn’s P = 0.023), indicating reduced TRG repertoire diversity in postnatally acquired infection (**Figure 4D**).

### 3.6 Specific clone comparison

**Figure 5:**
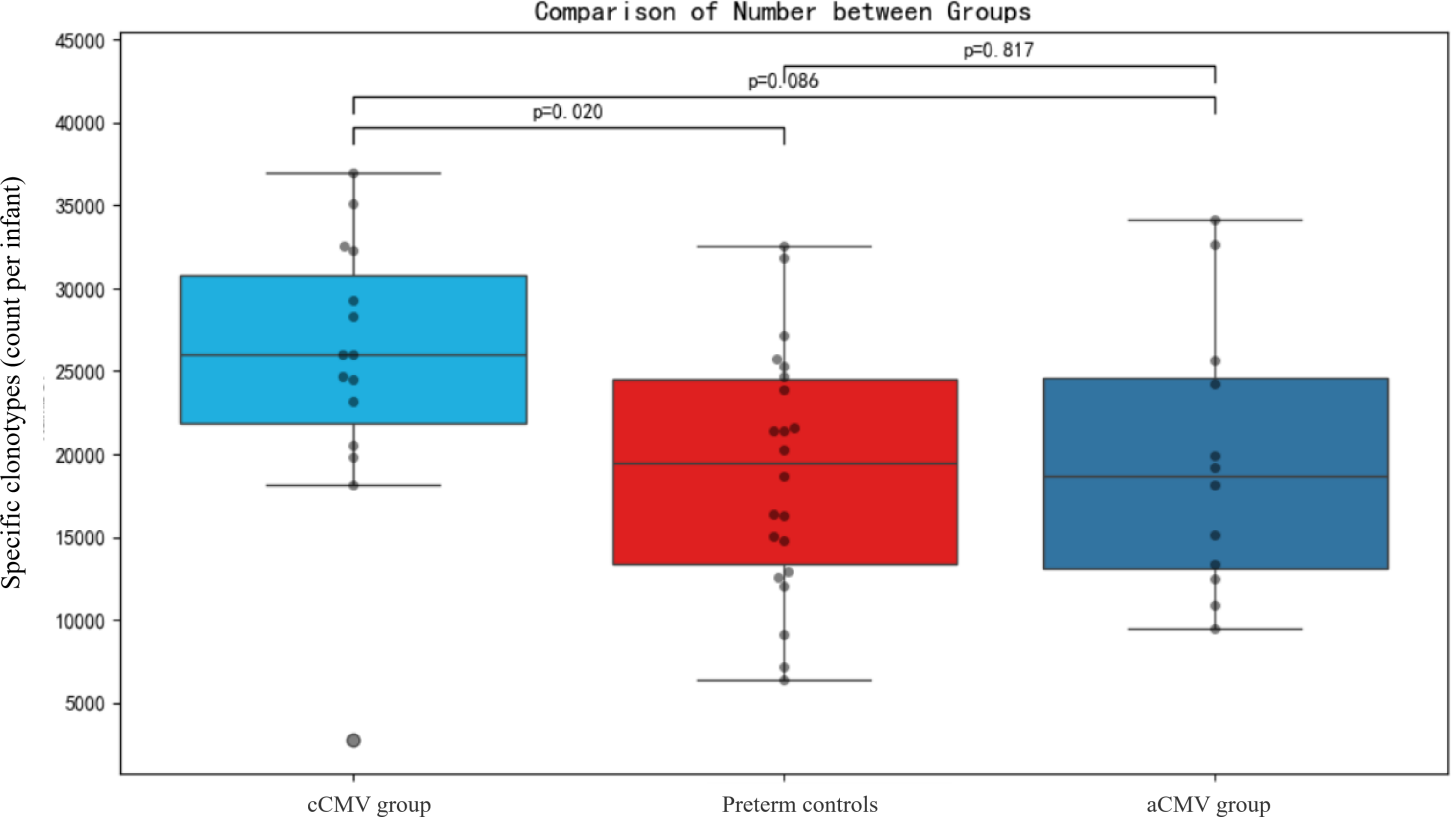
Sample-level counts of “specific” γδ TCR clonotypes across groups. Box-and-whisker plots show the per-infant count of “specific” clonotypes (definition in Methods) for the Congenital CMV infection (cCMV) group (n=9), Preterm controls (CMV-negative) (n=7), and the Acquired CMV infection (aCMV) group (n=10). Dots represent individual infants; boxes indicate the median and interquartile range; whiskers denote 1.5×IQR. Group comparisons used Kruskal–Wallis with Dunn’s *post-hoc* tests (FDR-adjusted): cCMV vs Preterm controls, p=0.020; Preterm controls vs aCMV, p=0.817; cCMV vs aCMV, p=0.086.

The number of “specific” clonotypes (defined as clonotypes detected exclusively in one group and absent in the other two) was significantly higher in the cCMV group (median = 26009.0, IQR: 21845.5–30761.5) than in preterm controls (median = 19446.0, IQR:13379.5–24481.8; Dunn’s P = 0.020; **Figure 5**). The cCMV group also showed a trend toward more specific clonotypes than the aCMV group, but this did not reach statistical significance (P = 0.086). No significant difference was observed between the aCMV group and preterm controls (P = 0.817).

### 3.7 Gene expression

**Figure 6:**
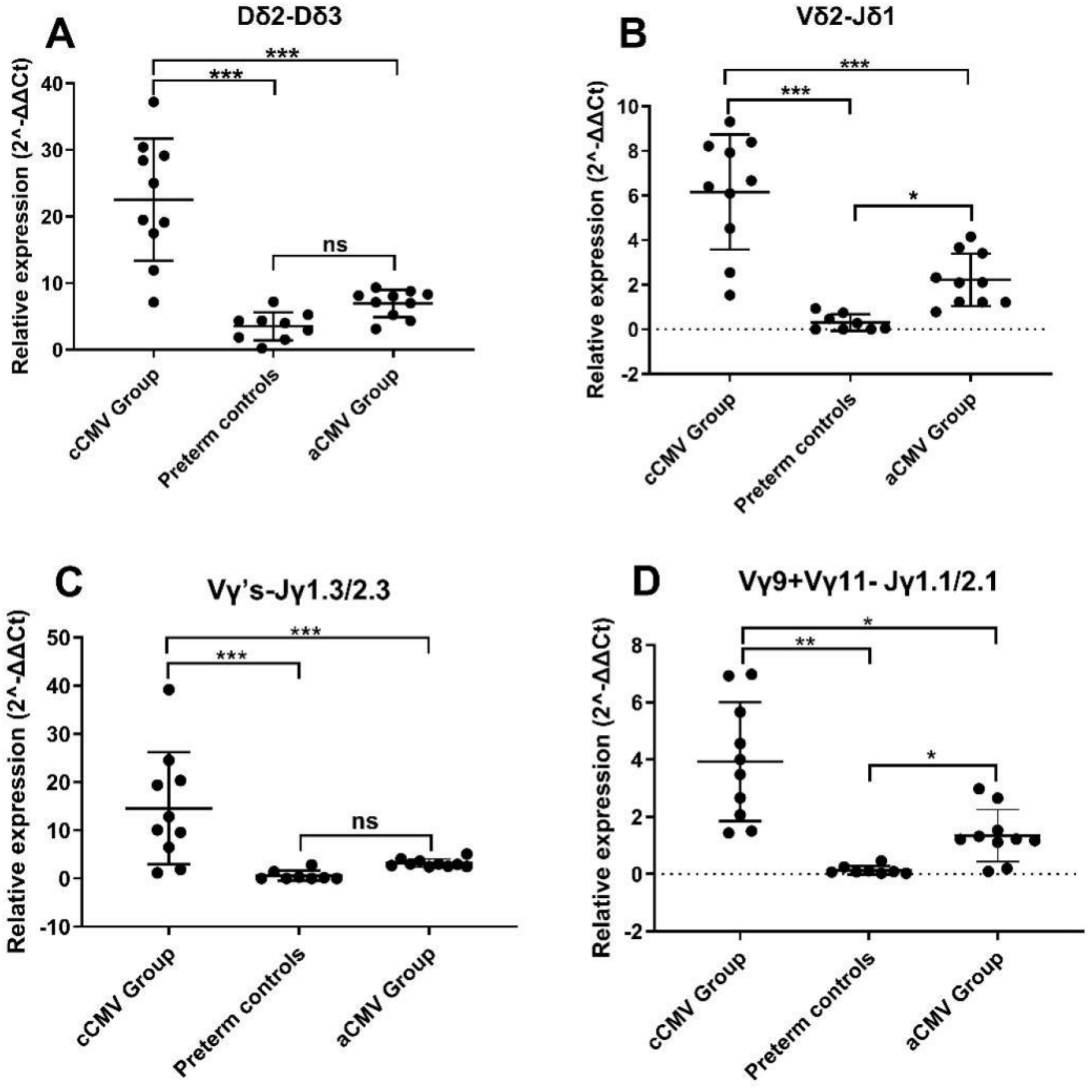
Relative expression of lineage-associated γδ T-cell rearrangements across groups. **(A)** Dδ2–Dδ3; **(B)** Vδ2–Jδ1; **(C)** TRGV1–Jγ1.3/2.3/Jγ1.2; **(D)** TRGV9+TRGV11–Jγ1.1/2.1 (mixed amplicon). SYBR Green–based qPCR normalized to GAPDH and expressed as 2^–ΔΔCt relative to Preterm controls (CMV-negative). Dots represent individual infants (cCMV group n=9; aCMV group n=10; Preterm controls n=7); bars show mean ± SD (or replace with median (IQR)). Brackets denote pairwise comparisons (Kruskal–Wallis with Dunn’s *post-hoc* tests, FDR corrected). Significance: ns, q≥0.05; *q<0.05; **q<0.01; ***q<0.001. **(D)** combines TRGV9 and TRGV11 forward primers; the assay does not separate Vγ9 from Vγ11 contributions.

We quantified nine lineage-associated rearrangement genes by SYBR Green–based qPCR with preamplification (**Table 1**). Expression of Dδ2–Dδ3 and Vδ2–Jδ1, which represent early thymic (δ chain) rearrangement intermediates, was significantly elevated in the cCMV group relative to preterm controls (Dδ2–Dδ3: fold change = [6.42], P = [<0.0001]; Vδ2–Jδ1: fold change = [20.04], P = [<0.0001]; **Figures 6A, B**), but did not differ between the aCMV group and controls. For the γ chain, expression of Vγ’s–Jγ1.3/2.3 was significantly higher in the cCMV group than in controls (fold change = [24.84], P = [<0.0001]; **Figure 6C**). The mixed Vγ9+Vγ11–Jγ1.1/2.1 amplicon was also elevated in the cCMV group (**Figure 6D**); however, because this assay combines TRGV9 and TRGV11 forward primers, it cannot disentangle the relative contributions of Vγ9 versus Vγ11, and deconvolution with Vγ-specific primers is warranted.

## 4 Discussion

This study investigated the γδ T-cell TCR repertoire from peripheral whole blood of preterm infants. It is important to note that peripheral blood predominantly captures circulating γδ T cell subsets—primarily Vδ2+ cells and, to a lesser extent, Vδ1+ cells—but under-represents tissue-resident populations. In humans, Vδ1+ γδ T cells are enriched in epithelial tissues such as the gut, skin, and liver, where they perform local immune surveillance (13). During CMV infection, tissue-resident Vδ1+ cells may expand locally before entering the circulation, meaning that peripheral blood sampling provides only a partial snapshot of the full γδ T cell response. This limitation is particularly relevant in neonates, in whom tissue γδ T cell seeding is still ongoing and compartmentalization is incomplete (13). Therefore, the repertoire alterations we describe may underestimate the full extent of γδ T cell remodeling induced by cCMV.

A central feature of the γδ T cell response to CMV is the selective expansion of Vδ2-negative populations, predominantly Vδ1+ cells, leading to a characteristic shift in the Vδ2+/Vδ2− ratio. This has been documented across transplant recipients, pregnant women, neonates, and healthy seropositive individuals (10). In the fetal setting, Vermijlen et al. demonstrated that CMV-driven expansion is restricted to Vγ9-negative γδ T cells, with a striking enrichment of a public Vγ8Vδ1 TCR bearing the germline-encoded CDR3δ1-CALGELGDDKLIF/CDR3γ8-CATWDTTGWFKIF (27). This public TCR was functionally validated as CMV-reactive and detectable from 21 weeks of gestation (27). Our observation that TRGV8 usage was elevated in the cCMV group is consistent with this paradigm and suggests that preterm infants with early CMV exposure mount a γδ T cell response bearing hallmarks of the fetal CMV response described by Vermijlen and colleagues.

Chain-specific repertoire remodeling. At the TRD chain level, the frequency of CDR3 sequences at the 25–amino-acid bin was lower in the Congenital CMV infection (cCMV) group than in Preterm controls (CMV-negative), and the cCMV group showed a lower Gini index than controls. Together, these findings indicate less clonal dominance and greater evenness in TRD among the cCMV group, arguing against a uniform oligoclonal-expansion model for cCMV at the δ chain and instead suggesting distributed recruitment of multiple TRD clonotypes (16). For the TRG chain, the cCMV group and the Acquired CMV infection (aCMV) group displayed length-interval–dependent shifts (e.g., the cCMV group lower at 12 aa but higher at 11 aa versus the aCMV group). Consistent with this, the Shannon index was lower in the aCMV group than in the cCMV group, indicating that postnatal infection may drive more focused expansions of selected TRG clonotypes, whereas *in-utero* exposure is associated with a broader, reshaped—rather than simply reduced—TRG repertoire.

V-gene usage and VJ pairing. We observed group-specific differences in V-gene usage: within TRD, usage of TRAV29/DV5 and TRAV38-2/DV8 differed in the cCMV group; within TRG, TRGV8 and TRGV9 showed distinct patterns across groups (e.g., TRGV8 higher in the cCMV group versus Preterm controls; TRGV9 higher in the aCMV group versus Preterm controls). These shifts suggest that cCMV selectively enriches V segments capable of recognizing cCMV-related antigens. The enrichment of TRGV8 in the cCMV group is of particular interest given the seminal finding by Vermijlen et al. that fetal CMV infection drives expansion of a public Vγ8Vδ1 TCR with antiviral activity (27). We screened our CDR3 data for the specific public clonotypes identified in that study (CDR3δ1-CALGELGDDKLIF and CDR3γ8-CATWDTTGWFKIF); The results showed that in TRD chain analysis, the frequency of the aCMV group was significantly higher than that of the cCMV group in the length intervals of 13 aa and 25 aa; The frequency of the aCMV group in the 25 aa interval was significantly higher than that of the preterm control group. In TRG chain analysis, the cCMV group has a higher frequency at 11 aa and a lower frequency at 12 aa; There was a significant difference in frequency between the cCMV group and the preterm control group at 12 aa; The frequency of the aCMV group was significantly different from that of the control group at 10 aa and 11 aa. This observation supports the concept that early-life CMV exposure elicits a conserved, germline-encoded γδ T cell response that may be shared across fetal and preterm populations. We also noted differences in VJ-pairing frequencies, with the cCMV group showing more frequent use of several VJ combinations than the aCMV group, consistent with antigen-driven selection of γδ TCRs (17).

Targeted rearrangements. Clone-level analyses showed more “specific” clonotypes in the cCMV group than in Preterm controls (CMV-negative), and intercalating dye–based qPCR assays detected higher expression for Dδ2–Dδ3, Vδ2–Jδ1, and Vγ–Jγ1.3/2.3 targets in the cCMV group. Signals were also elevated for the mixed Vγ9+Vγ11–Jγ1.1/2.1 amplicon. Because this assay combines TRGV9 and TRGV11 forward primers, it does not disentangle the relative contribution of Vγ9 versus Vγ11; therefore, deconvolution with Vγ-specific assays or repertoire sequencing is warranted (18). Taken together, these findings support antigen-experienced remodeling of γδ T cells in cCMV, while highlighting chain- and segment-specific differences between *in-utero* and postnatal infection.

Limitations and outlook. Several limitations of this study warrant consideration. First, the relatively small sample size, particularly in the cCMV group (n = 9), restricts statistical power for multivariable adjustments and subgroup analyses. Second, while we adhered to consensus guidelines defining congenital CMV (cCMV) as virological detection within the first 21 days of life (19, 20), this operational criterion cannot definitively exclude very early postnatal transmission, especially in preterm infants. Although postnatal transmission typically occurs later (incubation period: 30–120 days), early acquisition via breast milk has been documented in preterm infants as early as 4–7 weeks of age and occasionally earlier (21). To enhance transparency and allow readers to assess this risk, we have reported the Day of Life at first positive PCR for each subject in **Table 2**. Additionally, documented exposure to postnatal transmission sources—such as CMV-positive breastfeeding, blood transfusion—was incorporated as a supplementary criterion as the definition of the acquired CMV (aCMV) group, thereby enhancing diagnostic specificity in distinguishing congenital from postnatally acquired infection. Fourth,. Neonatal immune status is highly dynamic; gestational age, sampling time post-birth, intercurrent treatments (including anti-CMV therapy), and co-infections may confound repertoire readouts. Methodologically, our profiling quantifies expressed TCR transcripts using constant-region–anchored primers; thus it preferentially reflects activated/expanded clones rather than genomic rearrangement frequencies. The current protocol does not incorporate unique molecular identifiers (UMIs), which limits our ability to correct for PCR amplification bias. Multiplex primer competition (e.g., the Vγ9+Vγ11 amplicon) may also bias estimates. Furthermore, as our study profiled γδ T cells from peripheral blood only, tissue-resident γδ T cell populations (particularly Vδ1+ cells in gut, skin, and liver) were not captured. Future studies should (i) enlarge cohorts; (ii) separate Vγ9 from Vγ11 (and other overlapping primer sets) or apply UMI-based bulk/single-cell TCR-seq; (iii) incorporate functional readouts (cytokines, cytotoxicity) and phenotyping by flow cytometry; and (iv) model gestational age, sampling time, and therapy as covariates. Screening for the public Vγ8Vδ1 clonotypes described by Vermijlen et al. (27) in larger preterm cohorts would help establish whether these germline-encoded TCRs serve as reliable biomarkers of fetal CMV exposure. Ultimately, integrating V(D)J usage, CDR3-length signatures, and clone-level metrics into a molecular grading framework may improve risk stratification and guide targeted interventions for preterm infants with cCMV (22).

## Data Availability

The original contributions presented in the study are publicly available. This data can be found here: https://db.cngb.org/data_resources/?query=CNP0008876.
